# Dopant‐Free Crossconjugated Hole‐Transporting Polymers for Highly Efficient Perovskite Solar Cells

**DOI:** 10.1002/advs.201903331

**Published:** 2020-05-28

**Authors:** Xianglang Sun, Xiang Deng, Zhen Li, Bijin Xiong, Cheng Zhong, Zonglong Zhu, Zhong'an Li, Alex K.‐Y. Jen

**Affiliations:** ^1^ Key Laboratory for Material Chemistry of Energy Conversion and Storage Ministry of Education School of Chemistry and Chemical Engineering Huazhong University of Science and Technology Wuhan 430074 P. R. China; ^2^ Department of Chemistry City University of Hong Kong Kowloon 999077 Hong Kong SAR; ^3^ Department of Materials Science and Engineering City University of Hong Kong Kowloon 999077 Hong Kong; ^4^ Department of Chemistry Wuhan University Wuhan 430072 P. R. China

**Keywords:** crossconjugated polymers, dopant‐free hole‐transporting materials, perovskite growth, perovskite solar cells

## Abstract

Currently, there are only very few dopant‐free polymer hole‐transporting materials (HTMs) that can enable perovskite solar cells (PVSCs) to demonstrate a high power conversion efficiency (PCE) of greater than 20%. To address this need, a simple and efficient way is developed to synthesize novel crossconjugated polymers as high performance dopant‐free HTMs to endow PVSCs with a high PCE of 21.3%, which is among the highest values reported for single‐junction inverted PVSCs. More importantly, rational understanding of the reasons why two isomeric polymer HTMs (**PPE1** and **PPE2**) with almost identical photophysical properties, hole‐transporting ability, and surface wettability deliver so distinctly different device performance under similar device fabrication conditions is manifested. **PPE2** is found to improve the quality of perovskite films cast on top with larger grain sizes and more oriented crystallization. These results help unveil the new HTM design rules to influence the perovskite growth/crystallization for improving the performance of inverted PVSCs.

## Graphical abstract

Two isomeric crossconjugated polymer hole‐transporting materials (HTMs) are developed to demonstrate significantly distinct device power conversion efficiencies (PCEs) under the same device fabrication conditions, 11.1% **PPE1** and 19.3% for **PPE2**, which is found to be due to the improved quality of perovskite films made on top of **PPE2**. More excitingly, the **PPE2**‐based perovskite solar cells (PVSCs) can further achieve a more impressive PCE of 21.3% through suitable surface passivation.

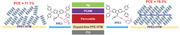

## 1 Introduction

In the past decade, the power conversion efficiency (PCE) of organic–inorganic hybrid perovskite solar cells (PVSCs) has increased rapidly from 3.8%^[^ ^1^ ^]^ to the certified 25.2%,^[^ ^2^ ^]^ showing very promising prospect as a new photovoltaic technology.^[^ ^3^, ^4^, ^5^, ^6^ ^]^ There are three commonly used device architectures for fabricating PVSCs,^[^ ^7^, ^8^, ^9^ ^]^ i.e., mesoscopic nanostructures, and conventional n–i–p and inverted p–i–n planar junction structures. Among these, inverted PVSCs have certain advantages such as suppressed photocurrent‐hysteresis and processing compatibility with flexible devices.^[^ ^10^, ^11^, ^12^, ^13^ ^]^ The first report of inverted PVSC was reported by Guo and co‐workers in 2013,^[^ ^14^ ^]^ affording a poor PCE of 3.9%. Subsequently, the PCEs of inverted PVSCs have been significantly improved to exceed 20% by applying new film‐deposition methods, optimizing device configurations, and proper interfacial engineering.^[^ ^15^, ^16^, ^17^, ^18^, ^19^ ^]^

For inverted p–i–n devices, a hole‐transporting layer (HTL) is deposited first, followed by a photoactive perovskite layer, and then an electron‐transporting layer (ETL). Thus, in this device architecture, HTL not only is responsible for transporting/extracting holes, but also plays an important role in affecting the growth of perovskite layer.^[^ ^20^, ^21^, ^22^, ^23^, ^24^ ^]^ Therefore, significant efforts have been devoted to regulating the surface properties of HTLs in inverted PVSCs to promote the formation of high quality perovskite films.^[^ ^22^, ^24^, ^25^, ^26^, ^27^, ^28^ ^]^ For example, Liao and co‐workers have introduced a perylene‐based interlayer between the perovskite layer and poly(3,4‐ethylenedioxythiophene)–polystyrenesulfonate (PEDOT:PSS) HTL to facilitate perovskite growth with enhanced crystallinity.^[^ ^27^ ^]^

To date, PEDOT:PSS and poly(bis(4‐phenyl)(2,4,6‐trimethylphenyl)amine) (PTAA) are the two most frequently used HTMs for inverted PVSCs.^[^ ^11^, ^12^ ^]^ PEDOT:PSS exhibits both a high hole conductivity due to its self‐doping ability and excellent wettability with perovskite precursor solutions. However, its inherent acidic and hygroscopic characteristics have been proven to hamper the long‐term stability of derived PVSCs, while the hydrophilic surface reduces the grain boundary mobility to limit grain sizes.^[^ ^29^, ^30^ ^]^ In addition, large potential loss is often found for the PEDOT:PSS‐based PVSCs due to mismatched work function between PEDOT:PSS and perovskites, which significantly limits their PCEs.^[^ ^11^, ^31^ ^]^

On the contrary, PTAA can produce much higher PCEs over 20%,^[^ ^17^, ^18^, ^19^, ^32^ ^]^ however, it is not only quite expensive, but also requires extra chemical doping procedures to achieve high performance.^[^ ^17^, ^24^, ^32^, ^33^, ^34^ ^]^ Unfortunately, chemical doping tends to degrade device performance because of the associated oxidation reactions.^[^ ^35^, ^36^, ^37^, ^38^ ^]^ Besides, its hydrophobic feature also increases the difficulty in coating perovskite films on top, especially for the roll‐to‐roll printing process.^[^ ^39^ ^]^ Thus, it is necessary to prewet the PTAA layer with solvent or apply an amphiphilic interlayer to facilitate the deposition of perovskites.^[^ ^18^, ^19^ ^]^ Hence, it is imperative to develop new high‐performance dopant‐free polymer HTMs with simple synthetic procedures and suitable surface wettability for inverted PVSCs,^[^ ^26^, ^35^, ^36^, ^37^, ^38^, ^40^, ^41^, ^42^, ^43^ ^]^ however the progress has been quite tardy with only very few dopant‐free polymer HTMs can afford PCE higher than 20% (Chart S1 and Table S1, Supporting Information).

**Figure 1:**
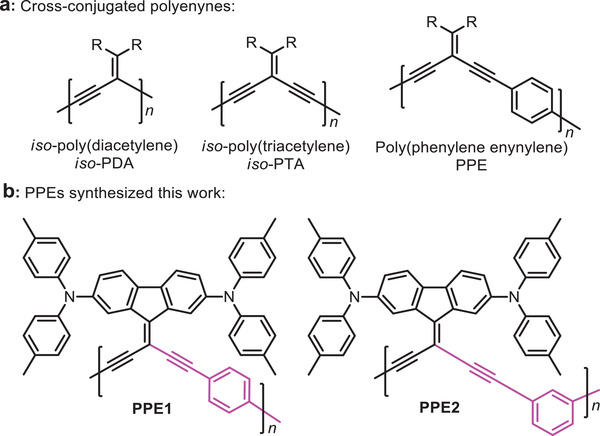
Structures of representative a) crossconjugated polyenynes and b) our designed **PPE1** and **PPE2** in this work.

Crossconjugated polymers have been demonstrated as an important class of organic semiconductors, due to their facile synthesis and interesting structural, physical, and optoelectronic properties.^[^ ^44^, ^45^, ^46^, ^47^, ^48^, ^49^ ^]^ Among them, particular interests have been paid on developing crossconjugated polyenynes, such as *iso*‐poly(diacetylene) (*iso*‐PDA) and *iso*‐poly(triacetylene) (*iso*‐PTA) (**Figure 1**a), possessing efficient *π*‐electron delocalization along the crossconjugated framework, although the extent is reduced compared to that of fully conjugated analogues.^[^ ^44^, ^50^, ^51^, ^52^ ^]^ Another attracting feature for crossconjugated polyenynes is their optical transparency in the visible region even with a relatively large *π*‐conjugation.^[^ ^53^, ^54^ ^]^ This is advantageous because it can help absorb damaging UV light and avoid competing with the perovskite light absorption.^19^ With the abovementioned characteristics, it is worth to explore crossconjugated polymer HTMs for their applications for PVSCs.

Herein, we report the facile synthesis of *iso*‐PTA derivatives as dopant‐free HTMs for highly efficient inverted PVSCs. In our design, a phenyl spacer is introduced onto the *iso*‐PTA backbone to form poly(phenylene enynylene)s (PPEs, Figure 1b), and by changing the linkage positions of phenyl spacers from para to meta, the backbone structure can be modified subtly to alter the crystallinity and optoelectronic properties of the resulting PPEs for investigating their influence on the perovskite properties and device performance. We further functionalize the vinylidene groups of PPEs by attaching 2,7‐diphenylamine capped fluorene units, one of the basic frameworks of dopant‐free HTMs,^[^ ^55^, ^56^, ^57^, ^58^ ^]^ to improve the hole mobility. Both PPEs possess proper energy levels, moderate hole mobilities, good surface wettability to the precursor solution of perovskites, and transparent window in the visible absorption region. Surprisingly, when they were used as dopant‐free HTMs for inverted PVSCs, the devices showed dramatically different PCEs, 11.13% for **PPE1** while 19.33% for **PPE2**, respectively, attributed to improved perovskite quality derived from **PPE2**. Furthermore, by rational surface passivation, the open circuit voltage (*V*_oc_) of **PPE2**‐based inverted PVSCs can be effectively improved to afford an impressive PCE of 21.3%.

## 2 Results and Discussion

**Scheme 1:**
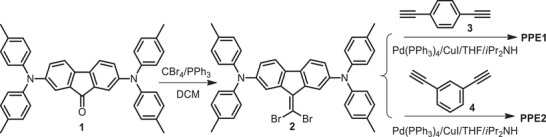
Synthetic route of crossconjugated polyenynes **PPE1** and **PPE2**.

The synthetic route for preparing PPEs is shown in **Scheme** **1**, which is quite straightforward comparing with those reported for donor–acceptor type polymer HTMs.^[^ ^59^, ^60^, ^61^, ^62^, ^63^, ^64^, ^65^, ^66^ ^]^ The synthetic details and characterization data are provided in Supporting Information. Compound **1** was synthesized from the reaction of two commercial raw materials according to our previous work,^[^ ^67^ ^]^ which can then be easily transferred to compound **2** via a Corey−Fuchs reaction at a good yield of 71%.^[^ ^68^ ^]^ The designed polymers **PPE1** and **PPE2** were obtained from compound **2** through a typical palladium‐catalyzed Sonogashira copolymerization with 1,4‐diethynylbenzene (**3**) and 1,3‐diethynylbenzene (**4**) to study the effect of para‐ and meta‐ conjugation on the final material properties. The copolymerized **PPE1** showed a much lower yield than that of **PPE2** (31.6% vs 86.1%), due to the formation of macrocycles by‐products. As a result, a much higher synthetic cost of $65.5 g^−1^ is calculated for **PPE1**, while that of **PPE2** is only $20.5 g^−1^ based on the research scale starting materials, which are much lower than that for PTAA.

**Figure 2:**
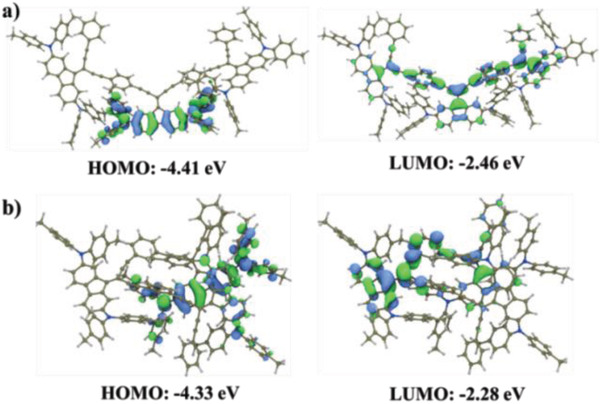
The DFT‐optimized molecular frontier orbitals of a) **PPE1** and b) **PPE2** based on the analogous trimer model.

The density functional theory (DFT)‐optimized structures are illustrated in Figure S1 (Supporting Information) based on the analogous trimer model, while the calculated highest occupied molecular orbital (HOMO) and lowest unoccupied molecular orbital (LUMO) energy levels are shown in **Figure** **2**. As shown, **PPE1** with paraconjugation shows a linear *π*‐conjugation but a high tendency to form macrocycles, thereby explaining its low copolymerization yield. On the contrast, **PPE2** with metaconjugation exhibits an unusual Zigzag polymer structure like typical polyenynes, containing a folded conformation with closer intramolecular stacking between the diphenylamine‐capped fluorene units. The torsional angles between two phenylacetylene groups and the central fluorene unit are also found to be quite different. For **PPE1**, it is similar with an angle of ≈45^o^, while for **PPE2**, there are two angles, 27^o^ and 68^o^, which could be the main reason for forming folded conformation. Both PPEs showed that the electron wave of their HOMOs are localizing on the diphenylamine‐capped fluorene units, while those of the LUMOs are delocalizing over the polymer backbone and partially extending to the fluorene units. The higher HOMO level for **PPE2** could be due to the strong intramolecular stacking between diphenylamine moieties, while the lower LUMO level for **PPE1** is possibly due to the para‐type linkage enables *π*‐conjugation to be more efficient.

Both PPEs have good solubility in organic solvents such as tetrahydrofuran (THF), chloroform (CF), chlorobenzene (CB), however, they can resist the erosion of *N,N*‐dimethylformamide (DMF) and dimethyl sulfoxide (DMSO), indicating the feasibility of processing perovskite precursor solutions on top of these films. The molecular weights of these two polymers were determined by gel permeation chromatography (GPC) using THF as the eluent, and the number‐average molecular weight (*M_n_*) of **PPE1** and **PPE2** were determined to be 10 800 and 11 900 g mol^−1^, respectively, with a similar polydispersity index (*Đ*) of 1.32, as listed in **Table** **1**. The onset thermal decomposition temperatures (*T*_d_s, 5% weight loss) were measured to be 460 and 414 °C for **PPE1** and **PPE2**, respectively, based on the thermogravimetric analysis (TGA, Figure S2, Supporting Information), suggesting they possess high thermal stability. Nevertheless, no clear glass transition temperatures and melting points could be observed in the differential scanning calorimetry (DSC, Figure S3, Supporting Information) for both PPEs.

**Table 1:** Molecular weights, thermal, optical, electrochemical, and charge transfer properties of **PPE1** and **PPE2**

HTM	*M_n_* [g mol^−1^]	*Đ*	*λ*_max_ ^a)^ [nm]		*E*_g_,_opt_ ^b)^ [eV]	*E*_HOMO_ ^c)^ [eV]	*E*_LUMO_ ^d)^ [eV]	*E*_HOMO_ ^e)^ [eV]	*T*_d_ ^f)^ [°C]	Mobility^g)^ [cm^2^ V^−1^ s^−1^]
			Solution	Film						
**PPE1**	10800	1.32	306, 384	383	2.45	−5.08	−2.63	−5.11	460	2.2 × 10^−6^
**PPE2**	11900	1.32	310, 385	384	2.70	−5.06	−2.36	−5.08	414	1.9 × 10^−6^

a) Absorption maxima;

b) Optical bandgaps calculated from film absorption edges;

c) Measured from electrochemistry experiments;

d) Calculated by subtracting *E*_g,opt_ from HOMO levels;

e) Measured from UPS experiments;

f) The 5% weight loss temperature detected by TGA under nitrogen;

g) Hole mobilties measured by SCLC method.

Both polymers have evident *π*–π* transition band at ≈385 nm, accompanied with a weak intramolecular charge transfer (ICT) band due to the electron‐deficient character of diacetylenephenyl groups (Figure S4, Supporting Information and Table 1). However, the ICT absorption band of **PPE1** is more red‐shifted than **PPE2**, owing to the more efficient paraconjugation. Moreover, it is worth to note that the absorption peak of PPEs at ≈320 nm, ascribed to the localized excitonic *π*−π* transition of diphenylamine units, disappeared from the solution state to the film state, which could be attributed to the enhanced intermolecular interactions.

**Figure 3:**
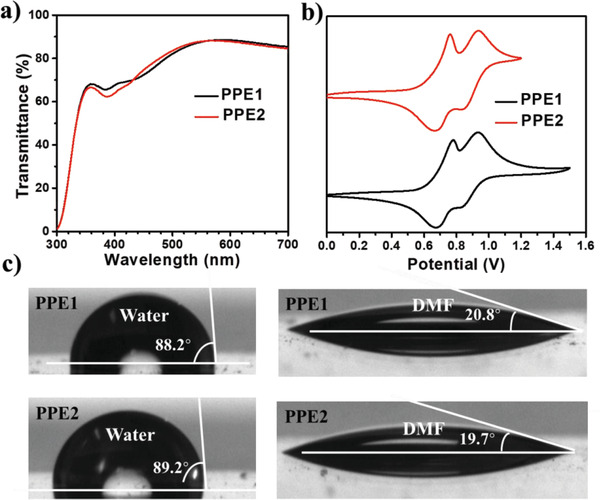
a) The film transmission spectra of polymer films on the ITO substrates. b) The CV curves of polymer films versus Fc/Fc^+^ (0.45 V) measured in CH_3_CN solutions. c) Contact angles of polymer films with respect to water and DMF drop.

Both PPEs also exhibit good film‐forming ability, and the resulting films (≈10 nm thick, Figure S5, Supporting Information) on indium tine oxide (ITO) substrates show uniform and smooth morphology with similar root‐mean‐square surface roughnesses (RMS) of 2.7 nm for **PPE1** and 2.5 nm for **PPE2**, respectively. For inverted PVSCs, good optical transparency of HTM is highly desirable because it can help absorb damaging UV light and avoid competing with the perovskite light absorption.^[^ ^41^, ^69^, ^70^ ^]^ In this regard, the transmission spectra of both polymer films on ITO are shown in **Figure 3**a, where both PPEs show similar transparency in the visible region.

The cyclic voltammetry (CV) curves of polymer films are shown in Figure 3b, with the related data listed in Table 1. The first average oxidation potentials (*E*_ox_) of **PPE1** and **PPE2** versus Fc/Fc^+^ (0.45 eV) were found to be 0.28 and 0.26 V, respectively, corresponding to the HOMO levels of −5.08 and −5.06 eV based on an equation of *E*_HOMO_ = −(*E*_ox_ + 4.80) eV. Moreover, the optical bandgaps (*E*_g,opt_s) of **PPE1** and **PPE2** films are calculated to be 2.45 and 2.70 eV, respectively, based on the film absorption onsets. By subtracting *E*_g,opt_s from the HOMO levels, the LUMO energy levels of **PPE1** and **PPE2** are estimated as −2.63 and −2.36 eV, respectively. To better understand their energy levels as films on ITO substrates, ultraviolet photoelectron spectroscopy (UPS, Figure S6, Supporting Information) was further employed to determine the HOMO levels of **PPE1** and **PPE2** to be −5.11 and −5.08 eV, respectively, which are consistent with those obtained from CV measurements. Overall, the suitable energy levels of both PPEs should be able to enable the hole transfer and the electron blocking when used as HTMs.

The surface wettability of polymer thin films was also evaluated as shown in Figure 3c. Both PPEs are found to have hydrophobic surface with a similar water contact angle of ≈90^o^, which could be beneficial for achieving large grain size crystals.^[^ ^24^ ^]^ They also have a comparable surface wettability to the polar DMF, the common solvent used for processing perovskites, with a contact angle of ≈20^o^. This suggests the feasibility of achieving good perovskite film formation with complete coverage on the PPE surface without the need of using prewetting or surface engineering, which is important for large area blade‐coating and roll‐to‐roll printing.^[^ ^39^ ^]^

A space‐charge‐limited‐current (SCLC) method was used to evaluate the hole mobilities of PPEs by fabricating hole‐only devices (Figure S7, Supporting Information), and both exhibit a moderate hole mobility of ≈2 × 10^−6^ cm^2^ V^−1^ s^−1^ (Table 1) compared to those reported for high‐performance dopant‐free HTMs.^[^ ^35^, ^36^, ^37^, ^38^ ^]^ Nonetheless, it is worth to note that the requirement for hole mobility can be relaxed somewhat for HTMs employed in the inverted PVSCs because only a very thin HTL is needed for realizing efficient hole extraction. For example, a thin fluorene‐cored small molecule HTM (≈10 nm) with a hole mobility of 2.18 × 10^−6^ cm^2^ V^−1^ s^−1^ was recently reported by Ding and co‐workers to show a high PCE of 19.06% in inverted PVSCs without adding any dopants.^[^ ^55^ ^]^

The molecular orientation of polymer films was studied by grazing incidence wide‐angle X‐ray scattering (GIWAXS, Figure S8, Supporting Information). As shown, the diffractions of both polymer films are oriented along an azimuthal angle of 45^o^ with respect to the substrate. Furthermore, the extracted scattering profiles for both PPEs in the out of plane direction show two strong peaks locating at *q* = 22 and 25 nm^−1^, respectively, suggesting the existence of two types of face‐on *π*–*π* stacking patterns with very close distances of ≈2.85 and ≈2.50 Å, respectively. These interesting packing behaviors are probably due to the particular crossconjugated polymer structure with large size of *π*‐conjugated side‐chains. Furthermore, we note that **PPE2** film exhibits higher diffraction intensity compared to **PPE1** film (Figure S8c, Supporting Information), thereby indicating an enhanced molecular crystallinity for the former, which is attributed to its unusual zigzag‐type polymer chain structure.^[^ ^71^, ^72^ ^]^ These results seem to be very contradictory by comparison with the hole mobility data in which **PPE1** shows a slightly higher mobility than **PPE2**. This discrepancy thus could be due to the limitation of SCLC measurement that emphasizes vertical charge transport.

**Figure 4:**
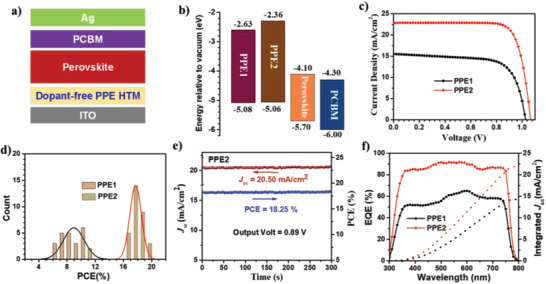
a) Device structure used in this study; b) corresponding energy levels relative to perovskite and PCBM; c) *J*–*V* curves of the champion PVSCs with PPEs as dopant‐free HTMs; d) Histograms of efficiency distributions of fabricated PVSCs; e) stable output current of **PPE2**‐based inverted PVSC under a constant bias of 0.89 V; f) EQE spectra with the integrated short‐circuit current density for champion PVSCs.

The inverted p–i–n planar PVSCs with a configuration of ITO/HTLs/perovskite/PCBM/Ag were fabricated to study the effectiveness of **PPE1** and **PPE2** as dopant‐free HTMs (**Figure 4**a). The energy‐level alignments of polymer HTMs relative to perovskites are shown in Figure 4b, indicating that both polymers could be used for hole extraction. The HTLs were processed by spin‐coating a CB solution (2 mg mL^−1^) of polymers on ITO without adding any dopants, followed by annealing at 150 °C for 10 min. (FAPbI_3_)_0.83_(MAPbBr_3_)_0.17_ (FA: formamidinium, MA: methylammonium) is used as the light‐absorber, in which a small amount of NH_4_BF_4_ is doped to improve the device performance according to our previous work.^[^ ^73^ ^]^ The device fabrication details are described in the Supporting Information.

The current density–voltage (*J*–*V*) curves of the champion dopant‐free inverted PVSCs measured under AM 1.5 G irradiation at 100 mW cm^−2^ are shown in Figure 4c, with relevant device parameters summarized in **Table** **2**. Interestingly, although **PPE1** and **PPE2** have a similar transparent window, HOMO energy levels and hole mobilities, they produce dramatically distinct device performance. **PPE1** only delivers a low PCE of 11.13% with a *V*_oc_ of 1.03 V, a short‐circuit current density (*J*_sc_) of 15.52 mA cm^−2^, and a fill factor (FF) of 69.54%, while for **PPE2**, a significantly enhanced PCE of 19.33% is achieved with a *V*_oc_ of 1.07 V, a *J*_sc_ of 22.84 mA cm^−2^, and a FF of 79.08%. The PCE histograms are provided in Figure 4d, showing very good reproducibility of our fabricated PVSCs with an average PCE of 9.5% for **PPE1** and 18.1% for **PPE2**, respectively.

**Table 2:** Device parameters of PVSCs using different polymer HTMs

HTM	*V*_OC_ [V]	*J*_SC_ [mA cm^−2^]	FF	PCE [%]
Dopant‐free **PPE1**	1.03 (1.02 ± 0.02)	15.52 (15.08 ± 0.65)	0.70 (0.69 ± 0.02)	11.13 (9.50 ± 1.06)
Dopant‐free **PPE2**	1.07 (1.06 ± 0.01)	22.84 (21.96 ± 0.83)	0.79 (0.77 ± 0.02)	19.33 (18.10 ± 0.83)
Dopant‐free **PPE2** ^a)^	1.18 (1.16 ± 0.02)	22.30 (22.17± 0.56)	0.81 (0.79 ± 0.02)	21.31 (19.77 ± 0.95)
Doped **PTAA** ^a)^	1.19 (1.17 ± 0.01)	22.65 (22.36 ± 0.77)	0.80 (0.79 ± 0.03)	21.56 (19.49 ± 1.30)

a) Devices treated with surface passivation by phenethylammonium iodide (PEAI).

Moreover, a stabilized PCE (18.25%) and *J*_sc_ (20.50 mA cm^−2^) can also be obtained for the **PPE2**‐based champion device when operated at the maximum power point (0.89 V), suggesting the high reliability of the *J*–*V* curves (Figure 4e). The external quantum efficiency (EQE) spectra (Figure 4f) were also collected, and the **PPE2**‐based device shows a much higher photo‐response throughout the entire spectrum from 300 to 800 nm compared to that from **PPE1**‐based device, confirming its much enhanced *J*_sc_. Furthermore, the integrated *J*_sc_ value is calculated to be 14.26 mA cm^−2^ for **PPE1** and 21.97 mA cm^−2^ for **PPE2**, respectively, close to those of the experimental values.

**Figure 5:**
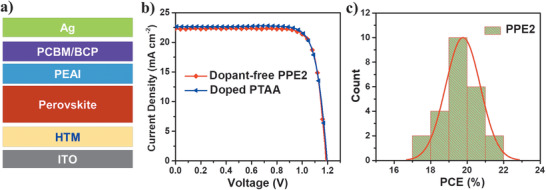
a) Device structure containing surface passivation layer of PEAI; b) *J*–*V* curves of the champion PVSCs based on dopant‐free PPE2 and doped PTAA HTMs; c) histograms of efficiency distributions of fabricated PVSCs based on dopant‐free **PPE2** HTM.

Previous studies have shown that surface defect passivation is an effective strategy to boost the device performance of PVSCs.^[^ ^74^ ^]^ As shown, although the device based on **PPE2** can achieve a respectable PCE of 19.33%, it is still limited by its relatively low *V*_oc_ (1.07 V) compared to those from the state‐of‐the‐art inverted PVSCs,^[^ ^15^, ^16^, ^17^, ^18^, ^19^ ^]^ which could be due to the misalignment between the HOMO levels of PPEs (≈−5.1 eV) and the valence band (VB) of the perovskite (≈−5.7 eV) as shown Figure 4b. Recently, Huang and co‐workers have demonstrated a very high *V*_oc_ of 1.23 V in inverted PVSCs by passivating the perovskite surface to suppress nonradiative recombination caused by the defects.^[^ ^19^ ^]^ Inspired by this work, we have also fabricated surface passivated PVSCs based on the **PPE2** HTM trying to further improve the device performance (**Figure 5**a). The PTAA‐based control devices doped with 2,3,5,6‐tetrafluoro‐7,7,8,8‐tetracyanoquinodimethane (F4‐TCNQ) were also fabricated for comparison. The *J*–*V* curves of champion devices are shown in Figure 5b, with related data listed in Table 2. An organic salt phenethylammonium iodide (PEAI) was used as the surface defect passivator, according to the report by You and co‐workers.^[^ ^75^ ^]^ The *V*_oc_ of PEAI passivated PVSCs is significantly improved from 1.07 to 1.18 V, without sacrificing any other device parameters, leading to a very impressive PCE of 21.31% with negligible current hysteresis (Figure S9, Supporting Information). This value is comparable to that from F4‐TCNQ‐doped PTAA based control devices (21.56%). Interestingly, we also found that **PPE2** exhibits a relatively high HOMO level compared to that of PTAA (≈−5.2 eV), but the resulting devices show a comparable *V*_OC_ with each other. This might be attributed to the change of the quasi‐Fermi level splitting (*V*_ap_) within the perovskites grown from different HTMs.^[^ ^76^, ^77^ ^]^ Furthermore, the PCE histogram of **PPE2**‐based devices (Figure 5c) also indicates good reproducibility with an average PCE of 19.77%. It is worth noting that the device PCE of 21.31% is among the best values achieved for dopant‐free HTMs, including small molecules and polymers,^[^ ^35^, ^36^, ^37^, ^38^ ^]^ and it is very close to the record‐high PCE (21.6%) reported for single‐junction inverted PVSCs.^[^ ^18^ ^]^ Combining with their low synthetic cost and proper wettability, our results demonstrate the great potential of using crossconjugated polymer HTMs for PVSCs.

**Figure 6:**
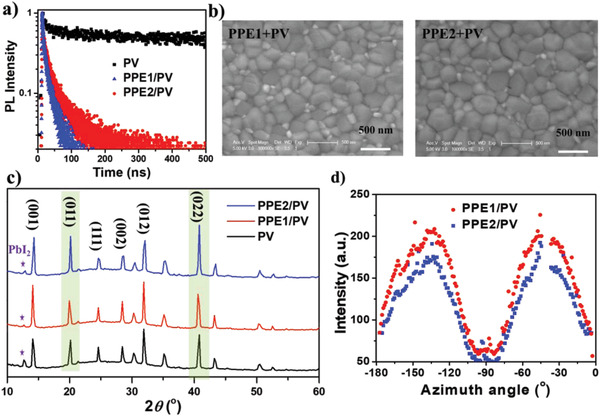
a) Time‐resolved PL spectra of bare perovskite (PV) film and bilayered PV films capped with dopant‐free polymer HTMs; b) SEM images of PV films atop PPEs; c) XRD patterns of bare PV film and bilayered PV films atop PPEs; d) The azimuthal intensity distributions for (100) plane in GIWAXS patterns of PV films atop PPEs. Note that the PV films were not passivated.

Given that PPEs are isomeric polymers with very similar optoelectronic properties, it would be interesting to understand the reason why such distinctly different device performance is obtained. To probe this, we try to investigate the effect of HTL itself by using perovskite films not passivated by PEAI. The hole extraction/transfer properties of fabricated devices were then investigated by collecting steady‐state photoluminescence (PL, Figure S10, Supporting Information) and time‐resolved PL spectra (**Figure 6**a) of the bi‐layered perovskite/dopant‐free HTM films. As shown, the PL of perovskites at ≈766 nm can be completely quenched when capping with the polymer HTMs, while the average PL decay time (*τ*) of bilayered films are also significantly shortened relative to that of bare perovskite film (≈1324 ns, Table S5, Supporting Information). Both indicate an efficient extraction of holes from the perovskite without the need of using any dopants. However, the *τ* for **PPE1** (24 ns) is found to be much shorter than that for **PPE2** (42 ns), while the **PPE2‐**based devices show much higher PCEs reversely. The common experience tells us faster decay time usually indicates more efficient hole extraction/transport and better suppressed charge recombination, which will result in higher device performance.^[^ ^78^, ^79^, ^80^, ^81^, ^82^, ^83^ ^]^ Therefore, there must be some other underlying factors related to the quality of perovskite film causing these controversial results. Indeed, for inverted PVSCs, the HTM morphology has been shown to play an important role in determining the growth of perovskite and its final film quality on top including grain sizes, crystal orientation, and defects.^[^ ^27^, ^28^ ^]^ Furthermore, the time‐resolved PL spectrum of the **PPE2**‐based passivated film was also measured, as shown in Figure S11 (Supporting Information) the *τ* greatly increases from 42 to 70 ns due to the PEAI passivation, further indicating the surface passivation of defects can effectively suppress the nonradiation recombination.

The surface morphology of the perovskite films was then investigated through the plane‐view scanning electron microscope (SEM, Figure 6b). It was found that the perovskite film atop of **PPE2** obviously exhibits larger grain sizes than that atop of **PPE1**. The crystallinity of perovskite films was further checked by studying their X‐ray powder diffraction (XRD) patterns. As shown in Figure 6c, all the characteristic crystallographic planes of perovskite can be observed, and the peak intensity of PbI_2_ is significantly decreased for perovskites made atop of polymer HTMs, meaning enhanced film quality. The residual signal of PbI_2_ in perovskite films on PPEs is attributed to the use of slightly excess PbI_2_ in preparing the perovskite films to reduce the defects.^[^ ^84^ ^]^ Encouragingly, the relative diffraction intensities of (011) and (022) crystallographic planes (Figure S12, Supporting Information) are much higher for perovskite film atop **PPE2**, comparing with those from **PPE1**. These results suggest a better quality of perovskite films produced atop of **PPE2**.

To assess the cause of different crystallinities in perovskite films, GIWAXS measurements were further conducted. As shown in Figure S13 (Supporting Information), the (100) and (200) crystal planes of resulting perovskites showed a *q* of 10 and 21 nm^−1^, respectively, indicating a preferred orientation along the azimuthal angle of 43° with respect to the substrate, similar to the reported results.^[^ ^85^ ^]^ In addition, both planes of perovskite films atop of **PPE2** show narrower azimuthal intensity distributions than those from **PPE1** (Figures 6d and Figure S14, Supporting Information), strongly suggesting a better oriented crystals for the former. Based on these results, it is reasonable to conclude that the significantly improved PCE for **PPE2** derived devices is due to its ability in enabling better quality perovskite films with more oriented crystals. This results in reduced charge traps and carrier recombination,^[^ ^86^ ^]^ thereby explains why the perovskite film atop of **PPE2** has a longer decay time than **PPE1**. Since both PPEs exhibit similar structure and properties, the most likely reason for the significant PCE difference could be due to the enhanced molecular crystallinity of **PPE2** in solid films as discussed above, enabling it as a potential template to facilitate the growth of ordered perovskite films with enhanced crystallinity through the van der Waals interactions between perovskite and HTL.^[^ ^22^, ^27^, ^28^ ^]^

## 3 Conclusion

In conclusion, we have developed a simple and effective way to synthesize two novel crossconjugated *iso*‐PTAs, **PPE1** and **PPE2**, by changing the linking positions of phenyl spacers from para to meta, in which 2,7‐diphenylamine capped fluorene units are attached to the vinylidene groups as side‐chains. By using these polymers as dopant‐free HTMs in inverted PVSCs, dramatically distinct PCEs of 11.13% for **PPE1** with paraconjugation and 19.33% for **PPE2** with metaconjugation were obtained. Through further application of PEAI to passivate the defects of perovskites, the *V*_oc_ of **PPE2**‐derived device was significantly improved from 1.07 to 1.18 V, affording a very impressive PCE of 21.31%. Given the fact that **PPE1** and **PPE2** are isomeric polymers with similar optical properties, surface wettability, energy levels, and hole mobilities, the significantly enhanced device performance of **PPE2**‐based PVSCs could be attributed to the enhanced perovskite quality grown atop its film. **PPE2** might serve as a good template to facilitate the growth of perovskites with larger grain sizes and more oriented crystals. This work provides a simple and effective way in developing high‐performance dopant‐free HTMs and highlights the critical role of HTL in affecting the perovskite growth/crystallization in inverted PVSCs.

## Conflict of Interest

The authors declare no conflict of interest.

## Supporting information

Supporting Information

## References

[1] A. Kojima , K. Teshima , Y. Shirai , T. Miyasaka , J. Am. Chem. Soc. 2009, 131, 6050. 19366264 10.1021/ja809598r

[2] Chart listing the best photovoltaic cell efficiencies, provided by NREL, https://www.nrel.gov/pv/assets/images/efficiency-chart.png (accessed: November 2019).

[3] M. A. Green , A. Ho‐Baillie , H. J. Snaith , Nat. Photonics 2014, 8, 506.

[4] P. K. Nayak , S. Mahesh , H. J. Snaith , D. Cahen , Nat. Rev. Mater. 2019, 4, 269.

[5] A. Rajagopal , K. Yao , A. K.‐Y. Jen , Adv. Mater. 2018, 30, 1800455. 10.1002/adma.201800455 29883006

[6] A. K. Jena , A. Kulkarni , T. Miyasaka , Chem. Rev. 2019, 119, 3036. 30821144 10.1021/acs.chemrev.8b00539

[7] H. S. Jung , N. G. Park , Small 2015, 11, 10.25358818

[8] L. Meng , J. You , T.‐F. Guo , Y. Yang , Acc. Chem. Res. 2016, 49, 155. 26693663 10.1021/acs.accounts.5b00404

[9] Y. Rong , Y. Hu , A. Mei , H. Tan , M. I. Saidaminov , S. I. Seok , M. D. McGehee , E. H. Sargent , H. Han , Science 2018, 361, eaat8235. 30237326 10.1126/science.aat8235

[10] P. Docampo , J. M. Ball , M. Darwich , G. E. Eperon , H. J. Snaith , Nat. Commun. 2013, 4, 2761. 24217714 10.1038/ncomms3761

[11] T. Liu , K. Chen , Q. Hu , R. Zhu , Q. Gong , Adv. Energy Mater. 2016, 6, 1600457.

[12] Y. Bai , X. Meng , S. Yang , Adv. Energy Mater. 2018, 8, 1701883.

[13] C. Zuo , H. J. Bolink , H. Han , J. Huang , D. Cahen , L. Ding , Adv. Sci. 2016, 3, 1500324. 10.1002/advs.201500324 PMC5066666 27812475

[14] J.‐Y. Jeng , Y.‐F. Chiang , M.‐H. Lee , S.‐R. Peng , T.‐F. Guo , P. Chen , T.‐C. Wen , Adv. Mater. 2013, 25, 3727. 23775589 10.1002/adma.201301327

[15] S. Yue , K. Liu , R. Xu , M. Li , M. Azam , K. Ren , J. Liu , Y. Sun , Z. Wang , D. Cao , X. Yan , S. Qu , Y. Lei , Z. Wang , Energy Environ. Sci. 2017, 10, 2570.

[16] K. Yao , S. Leng , Z. Liu , L. Fei , Y. Chen , S. Li , N. Zhou , J. Zhang , Y.‐X. Xu , L. Zhou , H. Huang , A. K. Y. Jen , Joule 2019, 3, 417.

[17] D. Luo , W. Yang , Z. Wang , A. Sadhanala , Q. Hu , R. Su , R. Shivanna , G. F. Trindade , J. F. Watts , Z. Xu , T. Liu , K. Chen , F. Ye , P. Wu , L. Zhao , J. Wu , Y. Tu , Y. Zhang , X. Yang , W. Zhang , R. H. Friend , Q. Gong , H. J. Snaith , R. Zhu , Science 2018, 360, 1442. 29954975 10.1126/science.aap9282

[18] M. Stolterfoht , C. M. Wolff , J. A. Márquez , S. Zhang , C. J. Hages , D. Rothhardt , S. Albrecht , P. L. Burn , P. Meredith , T. Unold , D. Neher , Nat. Energy 2018, 3, 847.

[19] S. Yang , J. Dai , Z. Yu , Y. Shao , Y. Zhou , X. Xiao , X. C. Zeng , J. Huang , J. Am. Chem. Soc. 2019, 141, 5781. 30888171 10.1021/jacs.8b13091

[20] B. Li , C. Zheng , H. Liu , J. Zhu , H. Zhang , D. Gao , W. Huang , ACS Appl. Mater. Interfaces 2016, 8, 27438. 27700051 10.1021/acsami.6b10342

[21] F. Galatopoulos , A. Savva , I. T. Papadas , S. A. Choulis , APL Mater.. 2017, 5, 076102.

[22] G. Tang , P. You , Q. Tai , A. Yang , J. Cao , F. Zheng , Z. Zhou , J. Zhao , P. K. L. Chan , F. Yan , Adv. Mater. 2019, 31, 1807689. 10.1002/adma.201807689 31033074

[23] C. Liu , J. Tu , X. Hu , Z. Huang , X. Meng , J. Yang , X. Duan , L. Tan , Z. Li , Y. Chen , Adv. Funct. Mater. 2019, 29, 1808059.

[24] C. Bi , Q. Wang , Y. Shao , Y. Yuan , Z. Xiao , J. Huang , Nat. Commun. 2015, 6, 7747. 26190275 10.1038/ncomms8747 PMC4518278

[25] J. Lee , H. Kang , G. Kim , H. Back , J. Kim , S. Hong , B. Park , E. Lee , K. Lee , Adv. Mater. 2017, 29, 1606363. 10.1002/adma.201606363 28394417

[26] Q. Xiao , F. Wu , M. Han , Z. Li , L. Zhu , Z. Li , J. Mater. Chem. A 2018, 6, 13644.

[27] Z.‐K. Wang , X. Gong , M. Li , Y. Hu , J.‐M. Wang , H. Ma , L.‐S. Liao , ACS Nano 2016, 10, 5479. 27128850 10.1021/acsnano.6b01904

[28] X. Zhao , L. Tao , H. Li , W. Huang , P. Sun , J. Liu , S. Liu , Q. Sun , Z. Cui , L. Sun , Y. Shen , Y. Yang , M. Wang , Nano Lett. 2018, 18, 2442. 29539264 10.1021/acs.nanolett.8b00025

[29] Q. Xue , G. Chen , M. Liu , J. Xiao , Z. Chen , Z. Hu , X.‐F. Jiang , B. Zhang , F. Huang , W. Yang , H.‐L. Yip , Y. Cao , Adv. Energy Mater. 2016, 6, 1502021.

[30] K.‐G. Lim , S. Ahn , Y.‐H. Kim , Y. Qi , T.‐W. Lee , Energy Environ. Sci. 2016, 9, 932.

[31] W. Yan , S. Ye , Y. Li , W. Sun , H. Rao , Z. Liu , Z. Bian , C. Huang , Adv. Energy Mater. 2016, 6, 1600474.

[32] W. S. Yang , B.‐W. Park , E. H. Jung , N. J. Jeon , Y. C. Kim , D. U. Lee , S. S. Shin , J. Seo , E. K. Kim , J. H. Noh , S. I. Seok , Science 2017, 356, 1376. 28663498 10.1126/science.aan2301

[33] Q. Wang , C. Bi , J. Huang , Nano Energy 2015, 15, 275.

[34] C.‐I. Chen , S. Wu , Y.‐A. Lu , C.‐C. Lee , K.‐C. Ho , Z. Zhu , W.‐C. Chen , C.‐C. Chueh , Adv. Sci. 2019, 6, 1901714. 10.1002/advs.201901714 PMC6839634 31728294

[35] W. Zhou , Z. Wen , P. Gao , Adv. Energy Mater. 2018, 8, 1702512.

[36] X. Sun , D. Zhao , Z. Li , Chin. Chem. Lett. 2018, 29, 219.

[37] K. Rakstys , C. Igci , M. K. Nazeeruddin , Chem. Sci. 2019, 10, 6748. 31391896 10.1039/c9sc01184f PMC6657418

[38] H. D. Pham , X. Li , W. Li , S. Manzhos , A. K. K. Kyaw , P. Sonar , Energy Environ. Sci. 2019, 12, 1177.

[39] W.‐Q. Wu , Q. Wang , Y. Fang , Y. Shao , S. Tang , Y. Deng , H. Lu , Y. Liu , T. Li , Z. Yang , A. Gruverman , J. Huang , Nat. Commun. 2018, 9, 1625. 29691390 10.1038/s41467-018-04028-8 PMC5915422

[40] W. Zhang , Y.‐C. Wang , X. Li , C. Song , L. Wan , K. Usman , J. Fang , Adv. Sci. 2018, 5, 1800159. 10.1002/advs.201800159 PMC6051387 30027048

[41] C.‐H. Tsai , N. Li , C.‐C. Lee , H.‐C. Wu , Z. Zhu , L. Wang , W.‐C. Chen , H. Yan , C.‐C. Chueh , J. Mater. Chem. A 2018, 6, 12999.

[42] D. Yang , T. Sano , Y. Yaguchi , H. Sun , H. Sasabe , J. Kido , Adv. Funct. Mater. 2019, 29, 1807556.

[43] L. Zhang , X. Zhou , X. Zhong , C. Cheng , Y. Tian , B. Xu , Nano Energy 2019, 57, 248.

[44] M. Gholami , R. R. Tykwinski , Chem. Rev. 2006, 106, 4997. 17165681 10.1021/cr0505573

[45] T. P. Voortman , D. Bartesaghi , L. J. A. Koster , R. C. Chiechi , Macromolecules 2015, 48, 7007.

[46] Y. Yao , H. Dong , F. Liu , T. P. Russell , W. Hu , Adv. Mater. 2017, 29, 1701251. 10.1002/adma.201701251 28585377

[47] Y. Zhang , H. Cheema , A. E. London , A. Morales , J. D. Azoulay , J. H. Delcamp , Phys. Chem. Chem. Phys. 2018, 20, 2438. 29308797 10.1039/c7cp06703h

[48] W. Zhang , Z. Mao , N. Zheng , J. Zou , L. Wang , C. Wei , J. Huang , D. Gao , G. Yu , J. Mater. Chem. C 2016, 4, 9266.

[49] G. W. P. Pruissen , J. Brebels , K. H. Hendriks , M. M. Wienk , R. A. J. Janssen , Macromolecules 2015, 48, 2435.

[50] Y. Zhao , R. R. Tykwinski , J. Am. Chem. Soc. 1999, 121, 458.

[51] Y. Zhao , R. McDonald , R. R. Tykwinski , J. Org. Chem. 2002, 67, 2805. 11975531 10.1021/jo015995y

[52] M. M. Rahman , X. Zhao , J. Harrell , L. Chen , A. Pietrangelo , ACS Macro Lett. 2017, 6, 632. 10.1021/acsmacrolett.7b00238 35650849

[53] S. C. Ciulei , R. R. Tykwinski , J. Org. Chem. 2000, 2, 3607. 10.1021/ol006491+ 11073656

[54] Y. Zhao , R. McDonald , R. R. Tykwinski , Chem. Commun. 2000, 77.

[55] J. Zhang , Q. Sun , Q. Chen , Y. Wang , Y. Zhou , B. Song , N. Yuan , J. Ding , Y. Li , Adv. Funct. Mater. 2019, 29, 1900484.

[56] H. D. Pham , L. Gil‐Escrig , K. Feron , S. Manzhos , S. Albrecht , H. J. Bolink , P. Sonar , J. Mater. Chem. A 2019, 7, 12507.

[57] Y. Zhang , C. Kou , J. Zhang , Y. Liu , W. Li , Z. Bo , M. Shao , J. Mater. Chem. A 2019, 7, 5522.

[58] J. Zhang , B. Xu , L. Yang , A. Mingorance , C. Ruan , Y. Hua , L. Wang , N. Vlachopoulos , M. Lira‐Cantú , G. Boschloo , A. Hagfeldt , L. Sun , E. M. J. Johansson , Adv. Energy Mater. 2017, 7, 1602736.

[59] H.‐C. Liao , T. L. D. Tam , P. Guo , Y. Wu , E. F. Manley , W. Huang , N. Zhou , C. M. M. Soe , B. Wang , M. R. Wasielewski , L. X. Chen , M. G. Kanatzidis , A. Facchetti , R. P. H. Chang , T. J. Marks , Adv. Energy Mater. 2016, 6, 1600502.

[60] G.‐W. Kim , G. Kang , J. Kim , G.‐Y. Lee , H. I. Kim , L. Pyeon , J. Lee , T. Park , Energy Environ. Sci. 2016, 9, 2326.

[61] K. Kranthiraja , K. Gunasekar , H. Kim , A. N. Cho , N. G. Park , S. Kim , B. J. Kim , R. Nishikubo , A. Saeki , M. Song , S. H. Jin , Adv. Mater. 2017, 29, 1700183. 10.1002/adma.201700183 28394431

[62] G.‐W. Kim , J. Lee , G. Kang , T. Kim , T. Park , Adv. Energy Mater. 2018, 8, 1701935.

[63] J. Lee , M. M. Byranvand , G. Kang , S. Y. Son , S. Song , G. W. Kim , T. Park , J. Am. Chem. Soc. 2017, 139, 12175. 28812350 10.1021/jacs.7b04949

[64] F. Qi , X. Deng , X. Wu , L. Huo , Y. Xiao , X. Lu , Z. Zhu , A. K. Y. Jen , Adv. Energy Mater. 2019, 9, 1902600.

[65] G. You , Q. Zhuang , L. Wang , X. Lin , D. Zou , Z. Lin , H. Zhen , W. Zhuang , Q. Ling , Adv. Energy Mater. 2020, 10, 1903146.

[66] Q. Xiao , J. Tian , Q. Xue , J. Wang , B. Xiong , M. Han , Z. Li , Z. Zhu , H.‐L. Yip , Z. a. Li , Angew. Chem., Int. Ed. 2019, 58, 17724. 10.1002/anie.201907331 31560144

[67] X. Sun , Q. Xue , Z. Zhu , Q. Xiao , K. Jiang , H. L. Yip , H. Yan , Z. Li , Chem. Sci. 2018, 9, 2698. 29732053 10.1039/c7sc05484j PMC5914136

[68] Z.‐Q. Chen , T. Chen , J.‐X. Liu , G.‐F. Zhang , C. Li , W.‐L. Gong , Z.‐J. Xiong , N.‐H. Xie , B. Z. Tang , M.‐Q. Zhu , Macromolecules 2015, 48, 7823.

[69] C. Huang , W. Fu , C. Z. Li , Z. Zhang , W. Qiu , M. Shi , P. Heremans , A. K. Jen , H. Chen , J. Am. Chem. Soc. 2016, 138, 2528. 26876042 10.1021/jacs.6b00039

[70] S. Ahmad , P. K. Kanaujia , W. Niu , J. J. Baumberg , G. Vijaya Prakash , ACS Appl. Mater. Interfaces 2014, 6, 10238. 24905435 10.1021/am501568j PMC4092025

[71] I. Osaka , T. Abe , S. Shinamura , E. Miyazaki , K. Takimiya , J. Am. Chem. Soc. 2010, 132, 5000. 20297819 10.1021/ja101125p

[72] S. Shi , X. Xie , P. Jiang , S. Chen , L. Wang , M. Wang , H. Wang , X. Li , G. Yu , Y. Li , Macromolecules 2013, 46, 3358.

[73] J. Zhang , S. Wu , T. Liu , Z. Zhu , A. K.‐Y. Jen , Adv. Funct. Mater. 29, 2019, 1808833.

[74] E. Aydin , M. De Bastiani , S. De Wolf , Adv. Mater. 2019, 31, 1900428. 10.1002/adma.201900428 31062907

[75] Q. Jiang , Y. Zhao , X. Zhang , X. Yang , Y. Chen , Z. Chu , Q. Ye , X. Li , Z. Yin , J. You , Nat. Photonics 2019, 13, 460.

[76] R. A. Belisle , P. Jain , R. Prasanna , T. Leijtens , M. D. McGehee , ACS Energy Lett. 2016, 1, 556.

[77] M. Stolterfoht , P. Caprioglio , C. M. Wolff , J. A. Márquez , J. Nordmann , S. Zhang , D. Rothhardt , U. Hörmann , Y. Amir , A. Redinger , L. Kegelmann , F. Zu , S. Albrecht , N. Koch , T. Kirchartz , M. Saliba , T. Unold , D. Neher , Energy Environ. Sci. 2019, 12, 2778.

[78] C. Shen , Y. Wu , H. Zhang , E. Li , W. Zhang , X. Xu , W. Wu , H. Tian , W.‐H. Zhu , Angew. Chem., Int. Ed. 2019, 58, 3784. 10.1002/anie.201811593 30701634

[79] S. Paek , P. Qin , Y. Lee , K. T. Cho , P. Gao , G. Grancini , E. Oveisi , P. Gratia , K. Rakstys , S. A. Al‐Muhtaseb , C. Ludwig , J. Ko , M. K. Nazeeruddin , Adv. Mater. 2017, 29, 1606555. 10.1002/adma.201606555 28714259

[80] F. Liu , F. Wu , Z. Tu , Q. Liao , Y. Gong , L. Zhu , Q. Li , Z. Li , Adv. Funct. Mater. 2019, 29, 1901296.

[81] Y. Wang , W. Chen , L. Wang , B. Tu , T. Chen , B. Liu , K. Yang , C. W. Koh , X. Zhang , H. Sun , G. Chen , X. Feng , H. Y. Woo , A. B. Djurišić , Z. He , X. Guo , Adv. Mater. 2019, 31, 1902781. 10.1002/adma.201902781 31292989

[82] X. Sun , F. Wu , C. Zhong , L. Zhu , Z. a. Li , Chem. Sci. 2019, 10, 6899. 31402973 10.1039/c9sc01697j PMC6640200

[83] X. Lai , F. Meng , Q.‐Q. Zhang , K. Wang , G. Li , Y. Wen , H. Ma , W. Li , X. Li , A. K. K. Kyaw , K. Wang , X. W. Sun , M. Du , X. Guo , J. Wang , W. Huang , Sol. RRL 2019, 3, 1900011.

[84] B.‐w. Park , N. Kedem , M. Kulbak , D. Y. Lee , W. S. Yang , N. J. Jeon , J. Seo , G. Kim , K. J. Kim , T. J. Shin , G. Hodes , D. Cahen , S. I. Seok , Nat. Commun. 2018, 9, 3301. 30120225 10.1038/s41467-018-05583-w PMC6098034

[85] L. Meng , C. Sun , R. Wang , W. Huang , Z. Zhao , P. Sun , T. Huang , J. Xue , J. W. Lee , C. Zhu , Y. Huang , Y. Li , Y. Yang , J. Am. Chem. Soc. 2018, 140, 17255. 30449094 10.1021/jacs.8b10520

[86] Z. Xu , Z. Liu , N. Li , G. Tang , G. Zheng , C. Zhu , Y. Chen , L. Wang , Y. Huang , L. Li , N. Zhou , J. Hong , Q. Chen , H. Zhou , Adv. Mater. 2019, 31, 1900390. 10.1002/adma.201900390 31012204

