# Supplementary material for: Dopant‐Free Crossconjugated Hole‐Transporting Polymers for Highly Efficient Perovskite Solar Cells
Source: Adv Sci (Weinh). 2020 May 28;7(13):1903331. doi: 10.1002/advs.201903331 (PMC7341082; doi:10.1002/advs.201903331)
Supplement: Supplementary file 1 — Supporting Information [file ADVS-7-1903331-s001.pdf]

Copyright WILEY-VCH Verlag GmbH & Co. KGaA, 69469 Weinheim, Germany, 2018.

Supporting Information

Dopant-free Crossconjugated Hole-Transporting Polymers for Highly Efficient Perovskite Solar Cells

Xianglang Sun†, Xiang Deng†, Zhen Li, Bijin Xiong, Cheng Zhong, Zonglong Zhu, Zhong'an Li* and Alex K.-Y. Jen**

Dr. X. Sun, Prof. B. Xiong, Prof. Z. A. Li
Key Laboratory for Material Chemistry of Energy Conversion and Storage, Ministry of Education, School of Chemistry and Chemical Engineering, Huazhong University of Science and Technology, Wuhan, 430074, P. R. China
Email: lizha@hust.edu.cn

X. Deng, Z. Li, Prof. Z. Zhu, Prof. A. K.-Y. Jen
Department of Chemistry, City University of Hong Kong, Kowloon, 999077, Hong Kong SAR
E-mail: zonglzhu@cityu.edu.hk; alexjen@cityu.edu.hk

X. Deng, Prof. A. K.-Y. Jen
Department of of Materials Science and Engineering, City University of Hong Kong, Kowloon, 999077, Hong Kong SAR

Prof. C. Zhong
Department of Chemistry, Wuhan University, Wuhan 430072, P. R. China

Instrumentations.

^{1}H NMR and ^{13}C NMR spectra were measured using a Bruker 400 or 600 MHz instrument spectrometer. High-resolution mass spectrometry (MS) was performed by using Bruker Daltonics instrument, SolariX 7.0T. Cyclic voltammetry was measured on a CHI6001 electrochemical analyzer (CH Instruments, Inc., China) using a conventional three-electrode cell with Pt metal as the working electrode, Pt gauze as the counter-electrode, and Ag/Ag^{+} as the reference electrode at a scan rate of 50 mV/s. Gel Permeation Chromatography (GPC) was used to investigate the molecular weight of polymers which was operated on Agilent PL-GPC 50 instrument. Thermal transition was measured on a NETZSCH STA 449 F3 Jupiter with a heating rate of 10 °C min^{-1}.

Differential scanning calorimetry (DSC) was recorded on a PerkinElmer 13 Diamond DSC in strument under nitrogen at a temperature scan of 10 °C/min. The contact angle was measured by KRÜSS DSA 25 contact angle goniometer. UV−vis−IR absorption spectra were collected using a Perkin Elmer UV-VIS-NIR spectrophotometer Lambda 750S. The ultraviolet photoelectron spectroscopy of HTLs were carried out in a VG ESCALAB 220i-XL surface analysis system equipped with a He-discharge lamp (hv = 21.22 eV). The atomic force microscopy (AFM) of HTLs was measured by using SHIMADZU SPM-9700SPM-9700 under the tapping mode. X-ray diffraction (XRD) characterization of perovskite layers was performed in a D2 Phaser instrument with a Cu Kα (λ=0.154 nm) radiation. The surface morphology of perovskite layers was acquired by scanning electron microscopy (SEM, Philips XL30 FEG). The steady-state photoluminescence (PL) and time-resolved PL spectra of the bi-layered perovskite/dopant-free HTM films were determined with a FLS980 spectrofluorometer from Edinburgh. All theoretical optimizations were done at B3LYP/def2-SVP level with Grimme's D3BJempirical dispersion correction, using Gaussian09 program.

Device fabrication and characterization.

ITO glass substrates were cleaned by sonication with detergent, deionized water, acetone and ethanol for 15 min, sequentially, which are then dried in the dry oven. The cleaned glass substrates were transferred into glove box for following film preparation. Solutions of **PPE1** and **PPE2** HTMs in chlorobenzene (CB, 2 mg/mL) were spin-coated on the ITO at 6000 rpm for 30 s as the HTLs. The fabricated HTLs were then thermally annealed on the hotplate at 150 ℃ for 10 min. Perovskite precursor solution was prepared by dissolving 171.9 mg FAI, 507.1 mg PbI_2, 22.4 mg MABr, 73.4 mg $PbBr_2$ in a 1 mL DMF/DMSO mixed solution (V/V, 5/1), in which 89 μL CsI solution (1.5 M in DMSO) and 1% in volume of NH_4BF_4 solution (1M in the mixed DMF/DMSO solution, (V/V, 5/1) were added. The perovskite solution was spin-coated on the HTL at 1000 rpm for 5 s and 6000 rpm for 30 s, respectively. Note that 110

μL of anti-solvent (CB) was quickly dropped at last 5 s of the film fabrication, followed by a thermal annealing at 100 °C for 30 min. Afterwards, PCBM solution (20 mg/mL in CB) was spin-coated on the perovskite layer at 1500 rpm for 40 s. Finally, 100 nm Ag electrode was evaporated under high vacuum. The device area is 10 mm^2.

For passivated devices, the processing of **PPE2** HTM and perovskite layer is similar to that described in above. The doped PTAA solution was prepared by adding 1 wt% F4-TCNQ (1mg/mL in CB) into PTAA solution (5 mg/mL in toluene), and then heated at 70°C overnight. Doped PTAA solution was spin-coated on the ITO substrates at 4000 rpm for 30 s, and then the samples were heated at 150 °C for 10 min. After depositing the perovskite layer onto the polymer HTMs (Dopant-free **PPE2** and doped PTAA), 100 μl of PEAI (2 mg/mL in IPA) was spin-coated onto the as-prepared perovskite films at the speed of 5000 rpm for 30s. The film then was heated at 100 °C for 10 min. Noted that the PEAI solution needed to be drop on the perovskite film quickly, and standing for 1-2 s before spin-coating. Then 35 μl $PC_{61}BM$ (20 mg/mL in CB) was spin coated on the top of perovskite film at 1500 rpm for 30 s. Finally, 6 nm BCP and 100 nm Ag electrode were evaporated under high vacuum. The device area is 10 mm^2.

The *J-V* characteristic curves were chacracterized under the AM 1.5G sunlight simulated by a simulator of Enlitech, SS-F5, Taiwan with a Keithley 2400 source meter. The steady-state output measurements were carried out without pre-illumination and UV filter. EQE spectra were collected with an EQE measurement system from EnLi Technology (Taiwan).

Synthetic Procedures

Materials.

Tetrahydrofuran (THF) and dichloromethance (DCM) were dried and distilled from sodium and anhydrous $CaCl_2$, respectively, under an atmosphere of dry nitrogen. Most of reagents

were purchased from Adamas (Titan Scientific, Shanghai), except $Pd(PPh_3)_4$ (Frontier). Compound **1** was synthesized as reported.[1]

Synthesis of monomer 2.

2,7-bis(di-p-tolylamino)-9H-fluoren-9-one (**1**, 1.00 g, 1.75 mmol), carbon tetrabromide (1.16 g, 3.50 mmol), and triphenyl phosphine (1.78 g, 6.79 mmol) were dissolved in 100 mL of anhydrous DCM. The reaction was run at 40 °C for 24 h, and the distilled water was added. The organic layer was collected, washed with water and dried with anhydrous Na_2SO_4. After concentration using a rotary evaporator, the crude product was purified by column chromatography on the silica gel using petroleum ether/DCM (V/V:10/1) as the eluent to obtain **2** as a red solid (0.90 g, 70.9%). ^{1}H NMR (400 MHz, Chloroform-*d*) δ 8.30 (d, *J* = 2.1 Hz, 2H, ArH), 7.36 (d, *J* = 8.2 Hz, 2H, ArH), 7.11 – 6.93 (m, 18H, ArH), 2.30 (s, 12H, $-CH_3$). ^{13}C NMR (101 MHz, Chloroform-*d*) δ 138.98, 132.28, 132.12, 132.02, 131.94, 129.81, 128.55, 128.43, 124.24, 121.27, 119.23, 90.83, 20.81. HRMS (APCI): $(M+H)^+$ = 725.1157 (calcd for $C_{42}H_{35}Br_2N_2^+$, 725.1162).

Synthesis of PPE1.

A mixture of compound **2** (0.40 g, 0.55 mmol), 1,4-diethynylbenzene (0.07 g, 0.55 mmol), CuI (0.03 g, 0.17 mmol) and $Pd(PPh_3)_4$ (26 mg, 0.04 mmol) in $i$$Pr_2NH$ (20 mL) and dry THF (40 mL) was heated to 70 ℃ under nitrogen for 72 h. Then the mixture was cooled to room temperature, extracted with DCM and washed with water. After concentrated, the solution was dropped into methanol, and the precipitation was filtrated and washed with methanol. Further purification of the crude products was conducted by exhaustive Soxhlet extraction with methanol (50 mL), acetone (50 mL), and hexane (50 mL) for 24 h successively. The product was collected after being dried under vacuum at 50 ℃ for 24 h and afforded as a yellow solid (0.12 g, 31.6%). ^{1}H NMR (400 MHz, Chloroform-*d*) δ 8.61 (br, 2H,

ArH), 7.38 (br, 2H, ArH), 7.10 - 6.79 (br, 18H, ArH), 2.20 (br, 4H, $-CH_3$). GPC: M_n = 10.8 kDa, Đ = 1.32.

Synthesis of PPE2.

A mixture of compound **2** (0.99 g, 1.36 mmol), 1,3-diethynylbenzene (0.17 g, 1.36 mmol), CuI (0.08 g, 0.41 mmol) and $Pd(PPh_3)_4$ (0.05 g, 0.04 mmol) in *i*Pr_2NH (36 mL) and dry THF (72 mL) was heated to 70 °C under nitrogen for 72 h. Then the mixture was cooled to room temperature, extracted with DCM and washed with water. After concentrated, the solution was dropped into methanol, and the precipitation was filtrated and washed with methanol. Further purification of the crude products was conducted by exhaustive Soxhlet extraction with methanol (50 mL), acetone (50 mL), and hexane (50 mL) for 24 h successively. The product was collected after being dried under vacuum at 50 °C for 24 h and afforded as a brown solid (0.81 g, 86.1%). ^1H NMR (400 MHz, Chloroform-*d*) δ 8.55 (br, 2H, ArH), 7.35 (br, 2H, ArH), 7.15 - 6.75 (br, 18H, ArH), 2.17 (br, 4H, $-CH_3$). GPC: M_n = 11.9 kDa, Đ = 1.32.

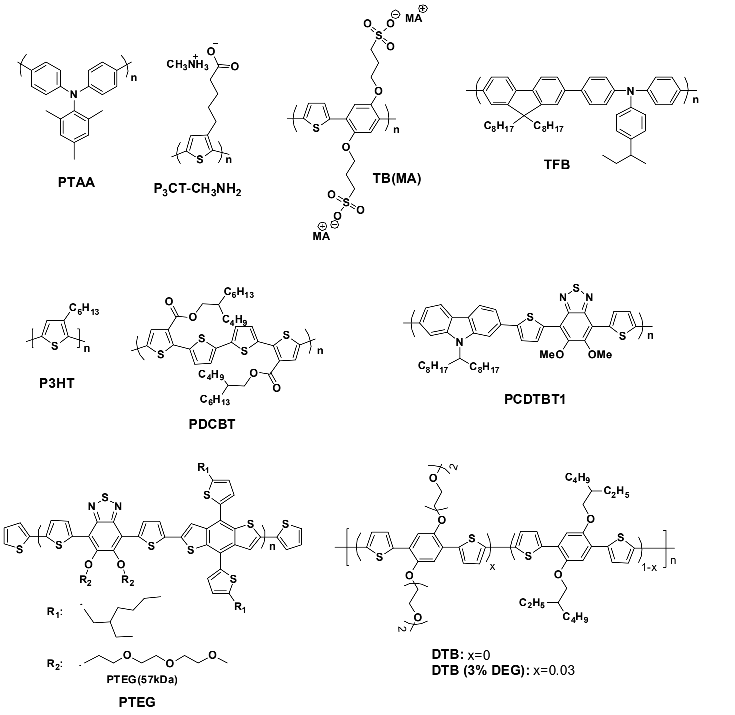

Chart S1. Structure of high performance dopant-free polymer HTMs report so far.

Table S1. The PCEs (>19%) of PVSCs based on dopant-free polymer HTMs.

HTM	PCE (%)	Device Structure	Reference
PTAA	21.6	p-i-n	*Nat. Energy* **2018**, *3*, 847.
PTAA	21.4	p-i-n	*J. Am. Chem. Soc.* **2019**, *141*, 5781.
PTAA	19.4	p-i-n	*Nat. Energy* **2016**, *1*, 15001
P3CT-CH_3NH_2	19.6	p-i-n	*ACS Appl. Mater. Interfaces* **2017**, *9*, 31357.
TB(MA)	19.76	p-i-n	*Nano Energy* **2019**, *57*, 248.
TFB	20.2	p-i-n	*Adv. Funct. Mater.* **2019**, *29*, 1807556.
P3HT	23.3	n-i-p	*Nature*, **2019**, *567*, 511.
PDCBT	21.2	n-i-p	*Science* **2017**, *358*, 1192.
PCDTBT1	19.1	n-i-p	*Nano Energy* **2018**, *45*, 28.
PTEG	19.8	n-i-p	*Adv. Energy Mater.* **2018**, *8*, 1701935
DTB	19.68	n-i-p	*Adv. Mater.* **2018**, *30*, 1804028
DTB(3%DEG)	20.19	n-i-p	*Adv. Funct. Mater.* 10.1002/adfm.201904856

Table S2. Synthetic cost for the synthesis of **2**

Chemical	Weight reagent (g/g, mL/g)	Weight solvent (mL/g)	Weight workup (g/g, mL/g)	Price of Chemical ($/Kg, $/L)	Chemical cost ($/g product)	Cost per step ($/step)
**1**	1.11			5970	6.62	
CBr_4 (adamas)	1.29			261	0.34	
PPh_3 (adamas)	1.98			75	0.15	
Na_2SO_4 (general source)			1.0	3.29	0.00	
CH_2Cl_2 (general source)			100.00	0.91	0.09	
Silica gel (general source)			50.00	8.73	0.44	
Petroleum ether (general source)			500.00	1.74	0.85	
						8.5

Table S3. Synthetic cost for the synthesis of **PPE1**

Chemical	Weight reagent (g/g, mL/g)	Weight solvent (mL/g)	Weight workup (mL/g)	Price of Chemical ($/Kg, $/L)	Chemical cost ($/g product)	Cost per step ($/step)
**2**	3.33			8490	28.27	
1,4-diethynylbenzene (Energy Chemical)	0.58			19074	11.06	
CuI (adamas)	0.25			495	0.12	
iPr_2NH (adamas)		166		20	3.32	
$Pd(PPh_3)_4$ (adamas)	0.22			6987	1.54	
THF (adamas)		333		32.27	10.74	
CH_2Cl_2(general source)			417	7.50	3.12	
methanol (general source)			417	8.73	3.64	
acetone (general source)			417	4.97	2.07	
hexane (general source)			417	3.90	1.63	
						65.5

Table S4. Synthetic cost for the synthesis of **PPE2**

Chemical	Weight reagent (g/g, mL/g)	Weight solvent (mL/g)	Weight workup (mL/g)	Price of Chemical ($/Kg, $/L)	Chemical cost ($/g product)	Cost per step ($/step)
**2**	1.22			8490	10.35	
1,3-diethynylbenzene (TCI)	0.21			20844	4.37	
CuI (adamas)	0.1			495	0.05	
iPr_2NH(adamas)		44		20	0.88	
$Pd(PPh_3)_4$ (adamas)	0.06			6987	0.42	
THF (adamas)		89		32.27	2.89	
CH_2Cl_2(general source)			62	7.50	0.46	
methanol (general source)			62	8.73	0.54	
acetone (general source)			62	4.97	0.31	
hexane (general source)			62	3.90	0.24	
						**20.5**

Table S5. Decay times extracted from time-resolved PL curves.

	τ_1 (ns)	τ_2 (ns)	average τ (ns)
**PPE1/PV**	8	40	24
**PPE2/PV**	11	72	42
**PV**	22	2626	1324

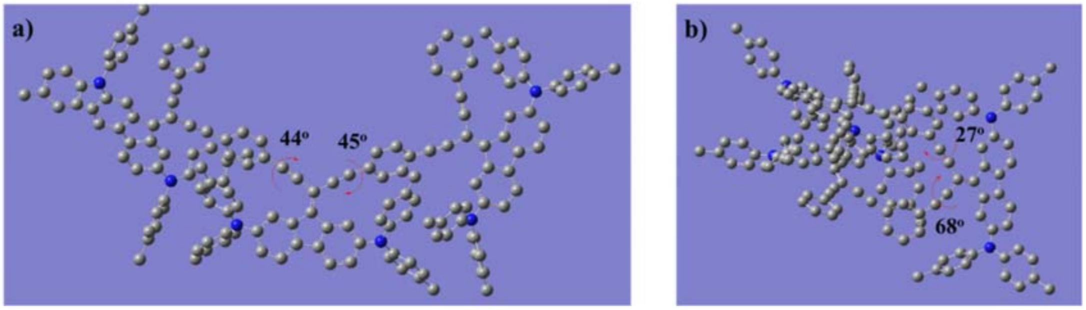

Figure S1. The DFT-optimized geometrical structure of **PPE1** and **PPE2**.

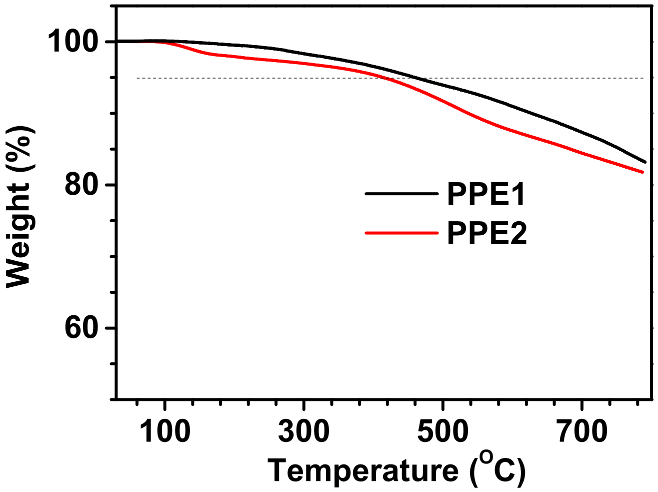

Figure S2. TGA curves of **PPE1** and **PPE2**.

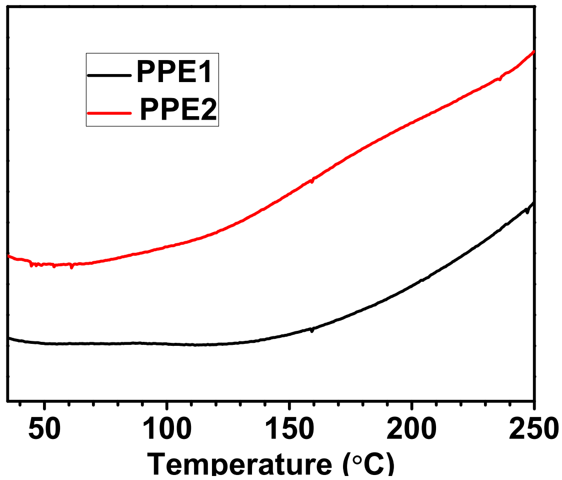

Figure S3. DSC curves of **PPE1** and **PPE2**.

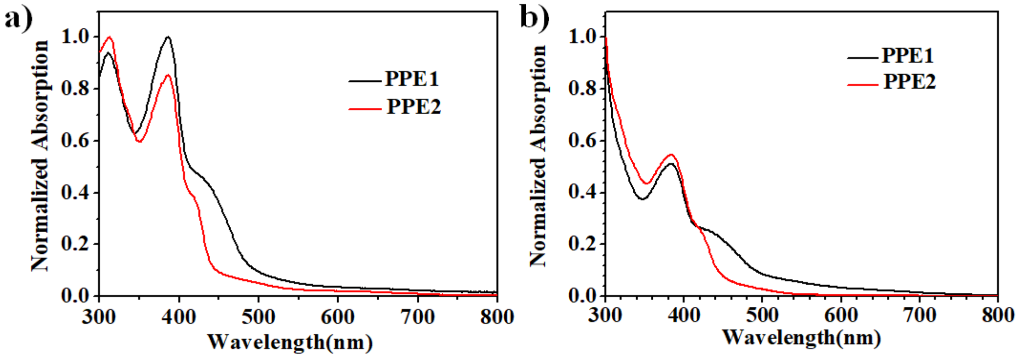

Figure S4. DCM solution (a) and thin film (b) absorption spectra of **PPE1** and **PPE2.**

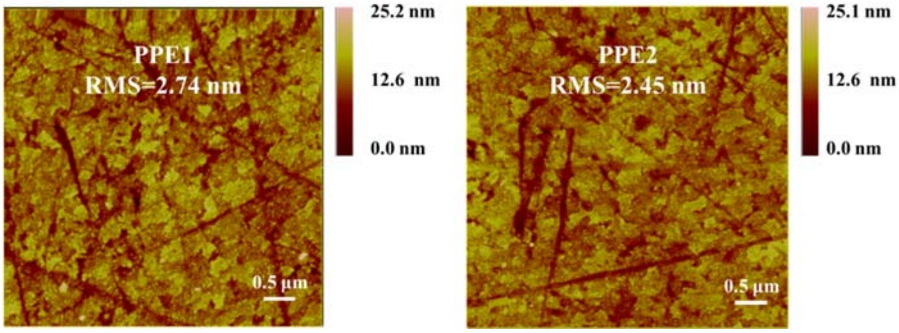

Figure S5. AFM images of **PPE1** and **PPE2** on the ITO substrates.

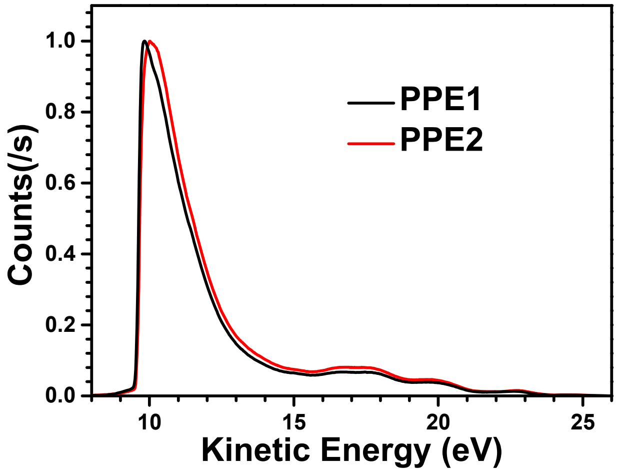

Figure S6. UPS of **PPE1** and **PPE2** films.

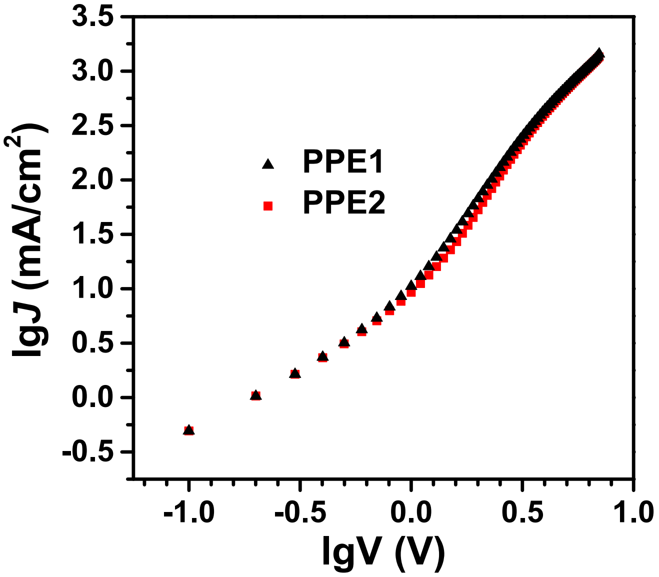

Figure S7. The hole injection characteristics of polymers measured by the SCLC method based on the device structure of ITO/PEDOT:PSS/HTMs/MoOx/Ag.

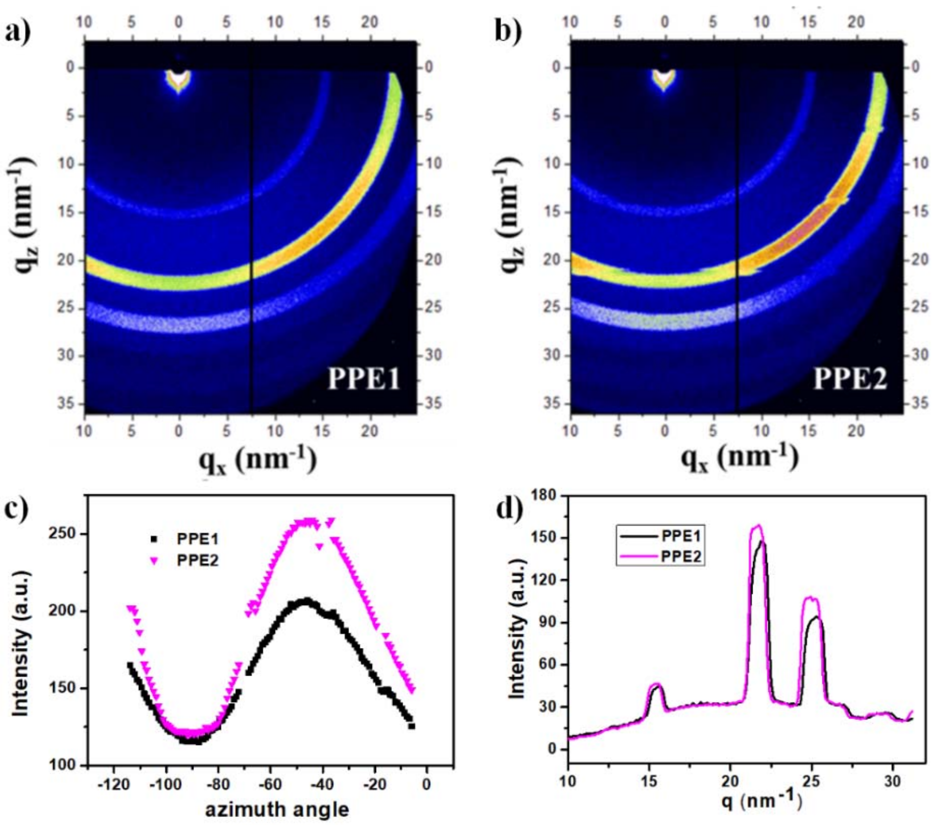

Figure S8. (a) The GIWAXS patterns of **PPE1** (a) and **PPE2** (b) films. (c) The azimuthal intensity distributions of **PPE1** and **PPE2** films**.** (d) Out-of-plane 1D GIWAXS patterns of **PPE1** and **PPE2** films.

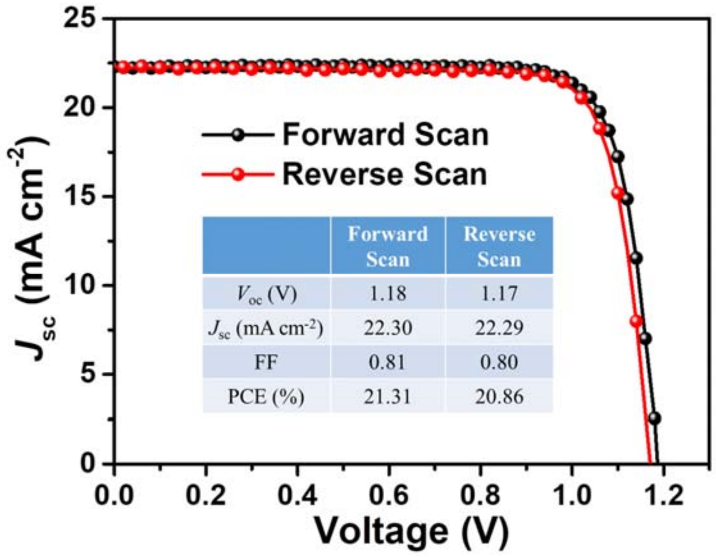

	Forward Scan	Reverse Scan
V_{oc} (V)	1.18	1.17
J_{sc} (mA cm^{-2})	22.30	22.29
FF	0.81	0.80
PCE (%)	21.31	20.86

Figure S9. *J*–*V* curves of the champion passivated PVSCs based on dopant-free **PPE2**.

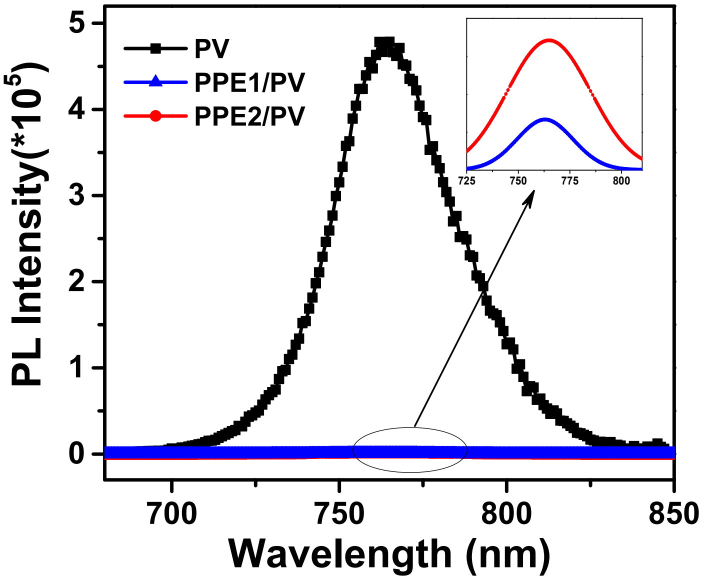

Figure S10. Steady PL spectra of bare perovskite (PV) films and bi-layered perovskite films capped with different dopant-free polymer HTMs. Note that the perovskite films were not passivated by PEAI.

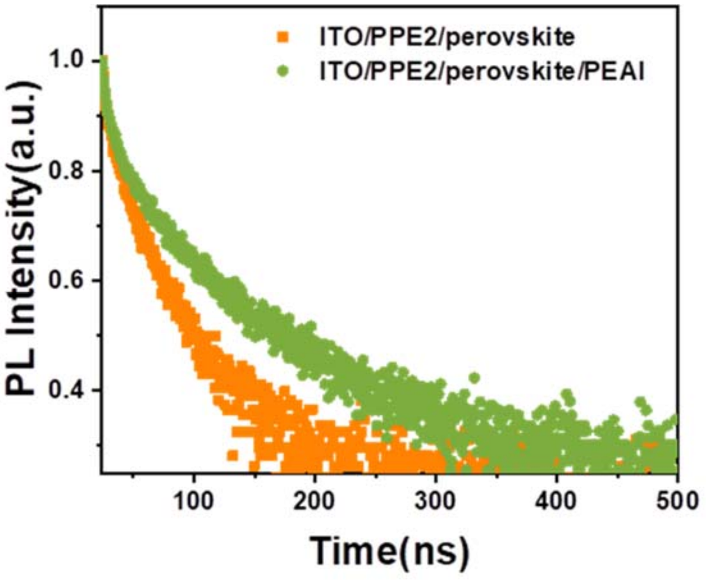

Figure S11. Time-resolved PL spectra of **PPE2**-based devices without and with PEAI passivation.

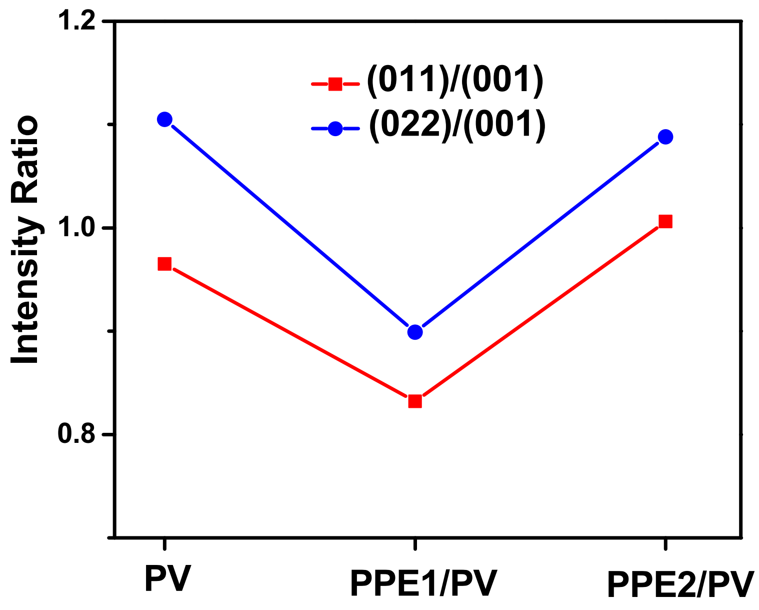

Figure S12. The relative diffraction intensities ascribed to (011) and (022) crystallographic planes in XRD patterns of perovskite films.

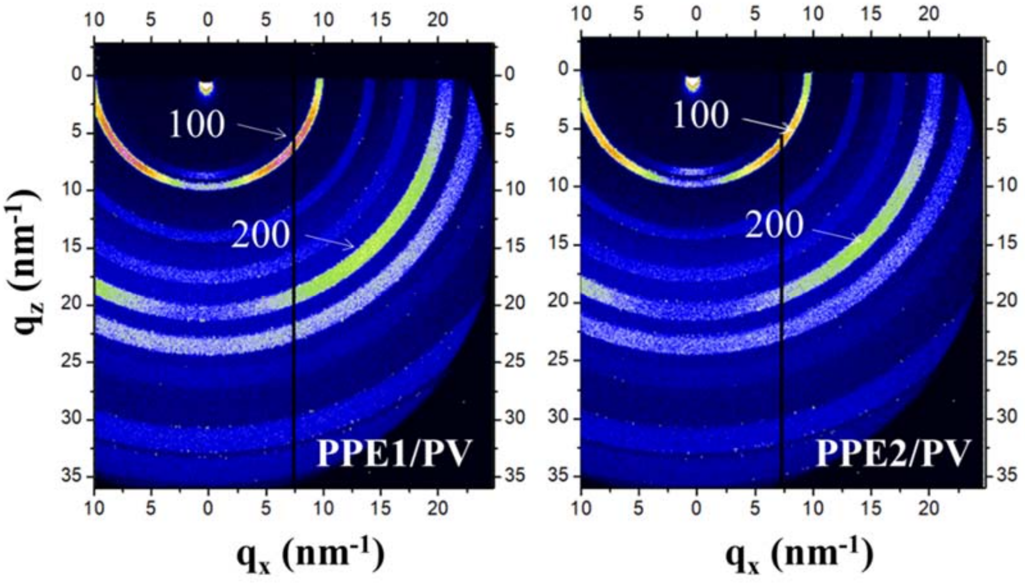

Figure S13. GIWAXS patterns of perovskite films atop PPEs.

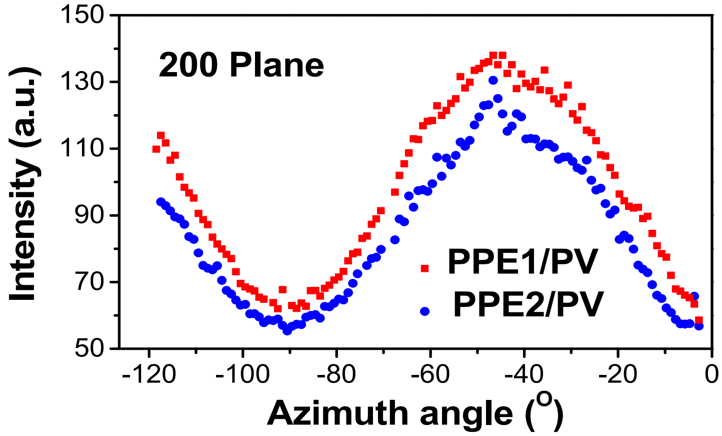

Figure S14. The azimuthal intensity distributions for (200) plane in GIWAXS patterns of perovskite films atop PPEs.

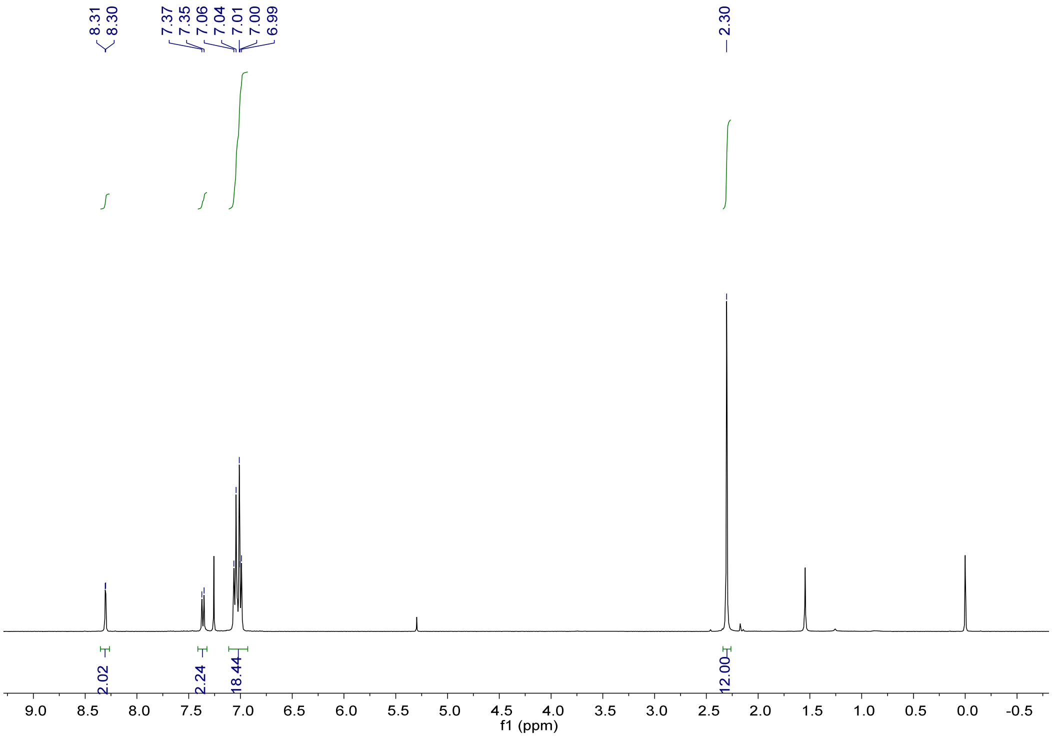

Figure S15. The ^{1}H NMR spectrum of monomer **2**, conducted in Chloroform-*d*.

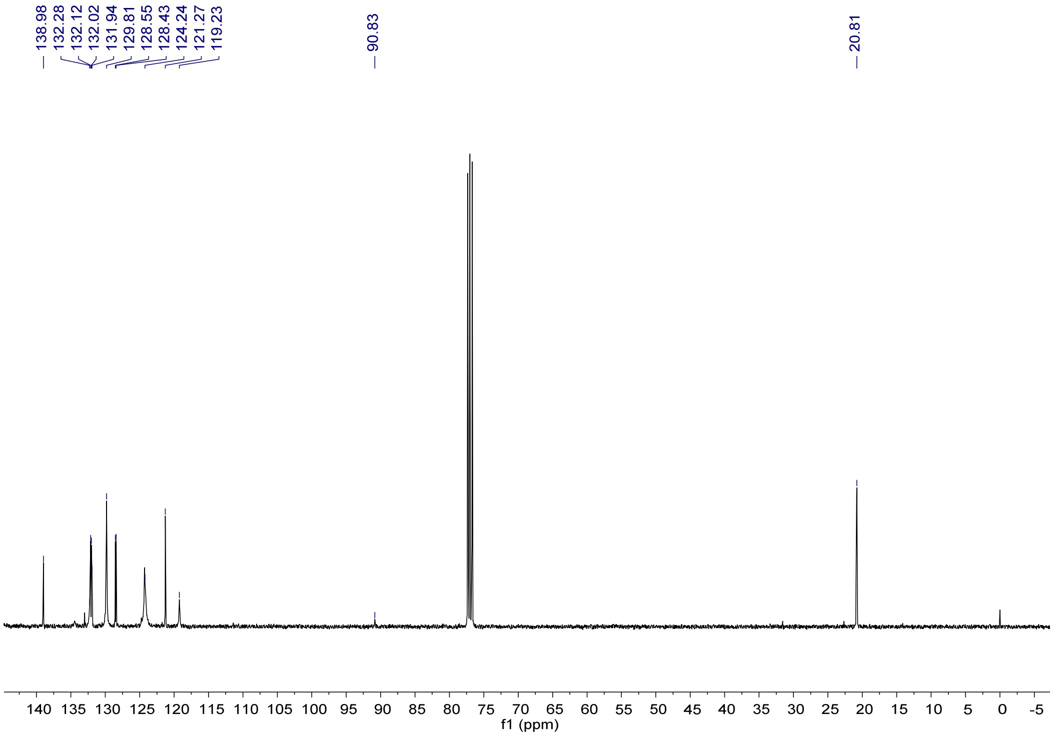

Figure S16. The ^{13}C NMR spectrum of monomer **2**, conducted in Chloroform-*d*.

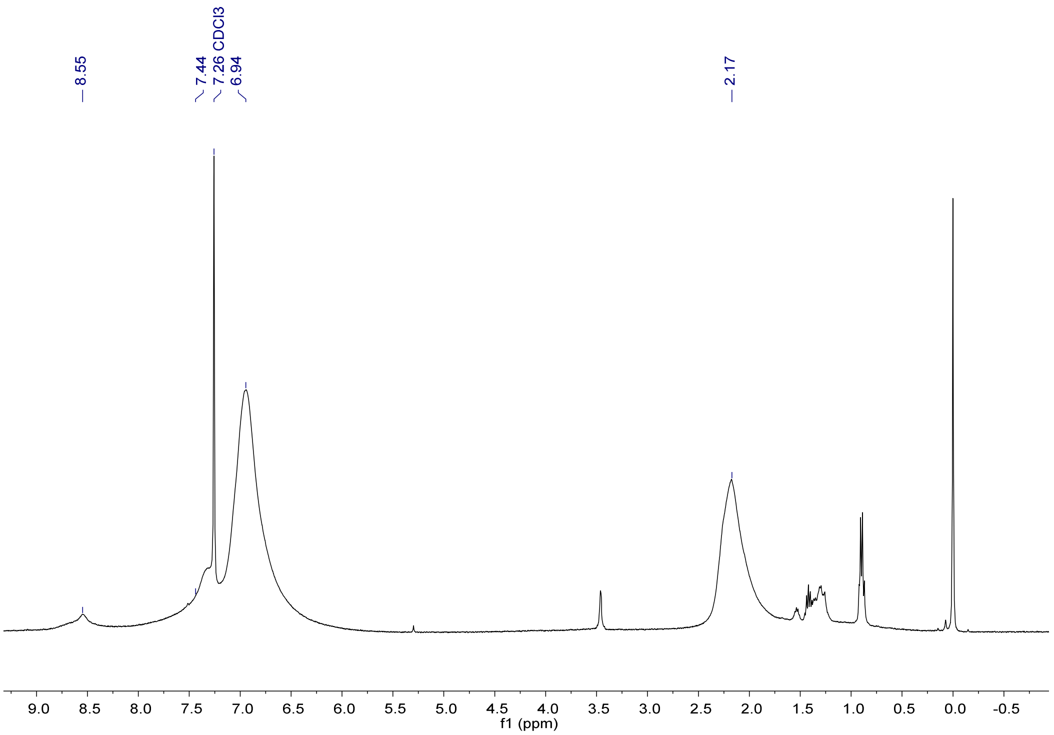

Figure S17. The ^{1}H NMR spectrum of **P1**, conducted in Chloroform-*d*.

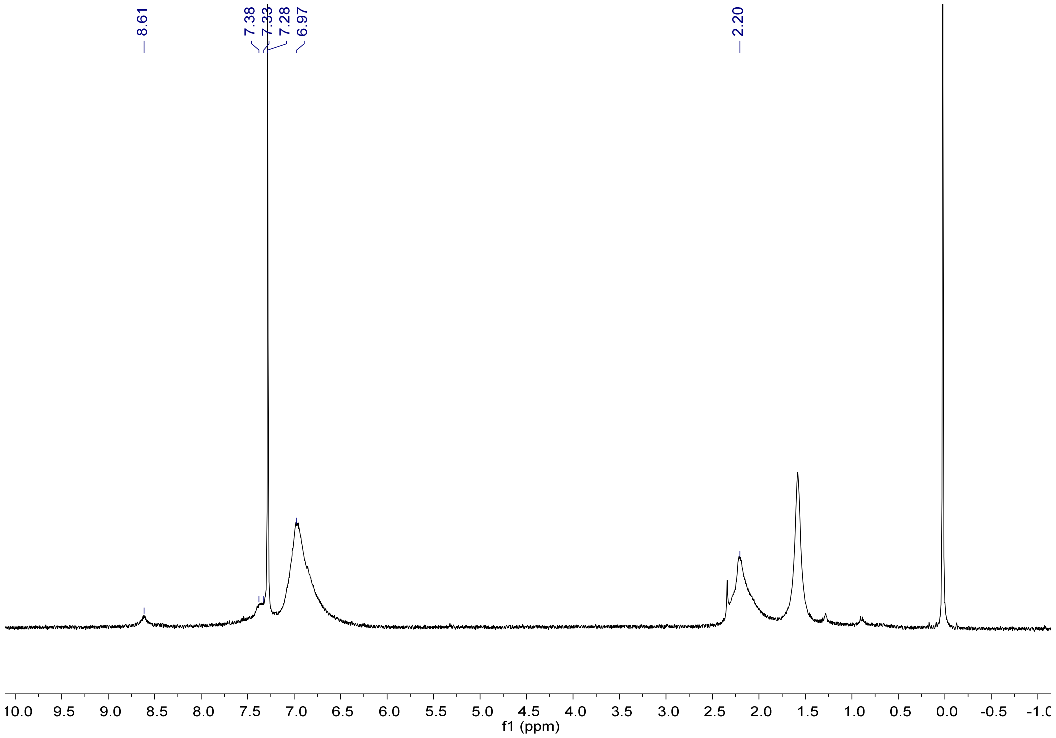

Figure S18. The ^{1}H NMR spectrum of **P2**, conducted in Chloroform-*d*.

Reference

1. X. Sun, Q. Xue, Z. Zhu, Q. Xiao, K. Jiang, H. L. Yip, H. Yan, Z. Li, *Chem. Sci.* **2018**, *9*, 2698.
